# Toxic metal phytoextraction potential and health-risk parameters of some cultivated plants when grown in metal-contaminated river sediment of Danube, near an industrial town

**DOI:** 10.1007/s10653-021-00880-8

**Published:** 2021-04-17

**Authors:** Éva Kovács-Bokor, Endre Domokos, Borbála Biró

**Affiliations:** 1 Institute of Engineering, University of Dunaújváros, Dunaújváros, Hungary; 2 Institute of Environmental Engineering, University of Pannonia, Veszprém, Hungary; 3 Department of Agroenvironmental Studies, Institute of Environmental Sciences, University of Szent István, Budapest, Hungary

**Keywords:** Potentially toxic elements, Sediment contamination, Edible plants, Phytoextraction, Health risk

## Abstract

Toxic metal phytoextraction potential of some higher plants, the white mustard (Sinapis alba L.)*,* perennial rye grass (Lolium perenne L.) and also two cultivated plants, as green pea (Pisum sativum L. var. Rajnai törpe), radish (Raphanus sativus L. var. Szentesi óriás vaj), was studied in a field experiment, along the river Danube in close vicinity of an industrial town, Dunaújváros, Hungary. Soil/sediment and the various plant organs (leaves, stems and roots) were assessed for the contamination with some potentially toxic elements (PTE), such as the cadmium (Cd), nickel (Ni), copper (Cu), and zinc (Zn). It was found that Cd and Ni concentration was below, while the Cu and Zn elements were above the Hungarian permissible limits in each of the studied soil/sediment samples. Bioconcentration factor (BAF) was less than 1 in the shoot biomass of test plant samples and followed the order of Cu > Zn > Cd and Ni. Phytoremediation potential of selected test plants was found to be rather limited. The translocation factor (TF) was more than 1 for Cu and Zn elements, at each test plants. Cadmium was translocated into the leaves in case of the radish, only. Considering of the potential human daily intake of metals (DIM), it was less than 1 both for the adults and for the children. Health risk index (HRI) values of children, however, were higher than 1 for the Cd in case of radish, and for Zn and Cu in case of the pea. Results suggest that consumption of these plants grown in gardens of contaminated sediments can result in some risks for citizens in the industrial town of Dunaújváros. Further studies are required to identify appropriate plants with greater toxic metal phytoextraction potential.

## Introduction

The river Danube is known to be the second longest river in Europe and the largest river in Hungary. The Hungarian section of this river is being 147 km. The depth, width and velocity of the river are rather fluctuating. The largest width in Budapest is 350 m the depth is between 3 and 10 m. The velocity is also changeable, the average is 0.5 m/s, but during flooding this value can be higher, 2.5 m/s (*vizugy.hu*). The river Danube is flowing across 8 countries, so it should be very important to keep of its water and sediment quality, in each of the targeted countries. Lots of industrial companies along the river might result in some risks, however, of reaching the permissible limits of the heavy metals and potentially toxic elements (PTE). The river Danube and its floodplain might be contaminated mainly by the nearby industrial power plants or by the industrial mining, similarly to other rivers (Chang et al. 2005; Mikanová et al. 2001; Šmuc et al. 2012). Most of the times, those waters and sediments are the primary recipients of the industrial- agricultural- and/or municipal pollutants (Dvorak et al. 2017).

Among the contaminants of surface waters or floodplain sediments, heavy metals are to be the most hazardous (Kovács-Bokor et al. 2019; Mikanová et al. 2001; Simon, 2014). These elements can show both the natural and the anthropogenic origin, so they might be ubiquitous in the environment (Khan et al. 2008). Natural sources can be for instance of the rock weathering and some of the volcanic activity. Anthropogenic emission is produced by mining, electroplating, agriculture (e.g. fertilisation) (Ali et al. 2013; Milenkovic & Damjanovic, 2005; Simon, 2014) or wastewater irrigation, solid waste disposal, sludge applications, vehicular exhaust (Khan et al. 2008) or other industrial by-products (e.g. different types of industrial sludge-types).

The effect of some potentially toxic elements on the environment could be rather serious because they are capable of bioaccumulation within the various segments of the plant-animal-human food-chain (Mónok & Kardos, 2018; Takács et al. 2001). Heavy metals and toxic elements are known to result serious damage in DNA chain with mutagenic or carcinogenic effects. Some of those pollutants furthermore can be persistent in soils and in sediments for rather long time. Heavy metals with known biological role in the soil–plant systems or being as essential elements, such as Cu, Zn, Ni can be easily taken up by the plants and can result in therefore potential risk. Non-essential elements, such as Cd, Cr, Pb, are generally non-accumulating in the plant-tissue, due to the fact, that higher plants are known to have several mechanisms avoiding of their uptake (Ali et al. 2013; Hooda, 2007)*.* In case of Cd for instance it was found, that test-plant of barley was developing a much larger root-system, so as to absorb the excess heavy metals in roots and avoiding the translocation towards the shoot biomass (Biró et al. 2005). Other solution for metal toxicity alleviation could be of using metal-adapted microorganisms, which are able to confer of their tolerance ability towards the higher plants and improving of a better survival even at highly toxic environments (Vivas et al. 2003).

Heavy metal accumulation in plants highly depends on plant species (Rattan et al. 2005)*.* The plants are capable of concentrating metals from the soils or sediments to roots or shoots (Biró et al. 2014; Ensley, 2000). Among the natural and cultivated plants, more than 400 species can be found to have phytoaccumulation potential. Various destructive and non-destructive measurements are known to study metal speciation in the soil–water-plant ecosystems (Kamnev et al. 2013). The hyperaccumulator plants are able to uptake metals in their shoots in a level of more than 1000 mg/kg in a dry weight basis (Baker & Brooks, 1989; Simon, 2006)*.* These plants belong mainly to the genus of *Brassicaceae, Alyssum* and *Thlaspi* regarding of the temperate zones (Hooda, 2007)*,* and *the main genus of Sebertia, Berkheya* at tropical zones (Baker et al. 1994). For example, *Thlaspi goesingense* and *Alyssum murale* species are hyperaccumulator plants for the Ni metal and can be used therefore for known phytomining activity (Bani et al. 2007; McGrath, 1998).

Among the contaminants, potentially toxic elements (PTE), generally called as “heavy metals” are in the focus of this study. Objective of this study was to investigate the metal contamination levels of the soil/sediment-plant system nearby of the river Danube along of an industrial town of Dunaújváros, Hungary. Other aim was to study metal uptake by some of the selected experimental plants, among them the food plants of radish (Raphanus sativus L. var. Szentesi óriás vaj) and green pea (Pisum sativum L. var. Rajnai törpe) and also the white mustard (Sinapis alba L.), and the perennial rye grass (Lolium perenne L.)*.* More particularly of their bioaccumulation potential was studied when grown on the soil/sediment substrates of the river Danube. During the field experiment Cd, Ni, Cu and Zn were assessed from soil/sediment samples and from the different parts (leaves, stems and roots) of available plant-biomass. Mobilisation potential of measured elements between sediment and test-plants was also determined, so as to get information about the phytoremediation potential and also the health risk potential of these cultivated plants, when grown on the targeted sites.

## Materials and methods

### Study area

Sampling sites were selected along the river Danube near the industrial town of Dunaújváros, Hungary. This town locates in the Transdanubian region of Hungary, approximately 80 km south from the Hungarian capital city, Budapest. Dunaújváros lies at the right side of the river Danube. In the town, several types of industrial factories are working e.g. the steel company of ISD-Dunaferr Ltd., the Hankook Tire Hungary Ltd., and also the paper industry by the Hamburger Hungaria Ltd. Many types of contaminants might be produced by these companies, although the sewage waters are cleaned, and the permissible limits of any contaminants are assessed and considered before reaching the effluents of the receiving water body. The final reservoir of the emitted sewage water is the river Danube.

The average heavy metal load of the river Danube and the study sites are represented by Tables 1 and 2. The average range of heavy metal concentrations in bottom sediments of the river Danube are 0.8–1.1 mg/kg for Cd, 15–50 mg/kg for Cr, 10–25 mg/kg for Cu, 20 mg/kg for Ni and 15–50 mg/kg for Pb (*JDS 3 2015*).

**Table 1:** The lowest (min.) and the highest (max.) values of some potentially toxic elements, assessed in the water, in suspended particle materials (SPM) and in the bottom sediment at the river Danube, as a main average in Hungary (*Joint Danube Survey 3, 2015*)

POTENTIALLY TOXIC ELEMENTS (PTE)	In water (µg/L)		In SPM^+^ (mg/kg dry weight)		In bottom sediment (mg/kg dry weight)	
	Min	Max	Min	Max	Min	Max
Cadmium (Cd)	< 0.01	0.145	0.26	1.16	0.72	1.95
Chromium (Cr)	0.29	6.73	37.0	76.1	9.8	101.1
Copper (Cu)	1.06	9.93	26.8	86.7	< LOQ^+^	105.0
Nickel (Ni)	0.78	24.63	29.0	69.3	5.1	9.2
Lead (Pb)	0.2	8.08	< 18.0	48.7	13.7	85.0
Zinc (Zn)	1.13	12.95	99.5	245.6	23	198

^**+**^LOQ = Limit of quantification

**Table 2:** Potentially toxic elements (PTE) in water, in suspended particle materials (SPM) and in bottom sediment of the river Danube at Dunaföldvár Bridge, Hungary

Potentially toxic elements (PTE)	In water (µg/L)	In SPM^+^ (mg/kg dry weight)	In bottom sediment (mg/kg dry weight)
Cadmium (Cd)	0.036	0.4	0.65
Chromium (Cr)	NDA^+^	NDA^+^	NDA^+^
Copper (Cu)	NDA^+^	NDA^+^	NDA^+^
Nickel (Ni)	1.5	33	10
Lead (Pb)	0.3	22	18
Zinc (Zn)	NDA^+^	NDA^+^	NDA^+^

The samples were collected at the 1560 flow km of the river. JDS24 is the identification number of this sampling place of Dunaföldvár Bridge in JDS 3 (*Joint Danube Survey 3, 2015*)

^+^NDA = No data available

**Fig. 1:**
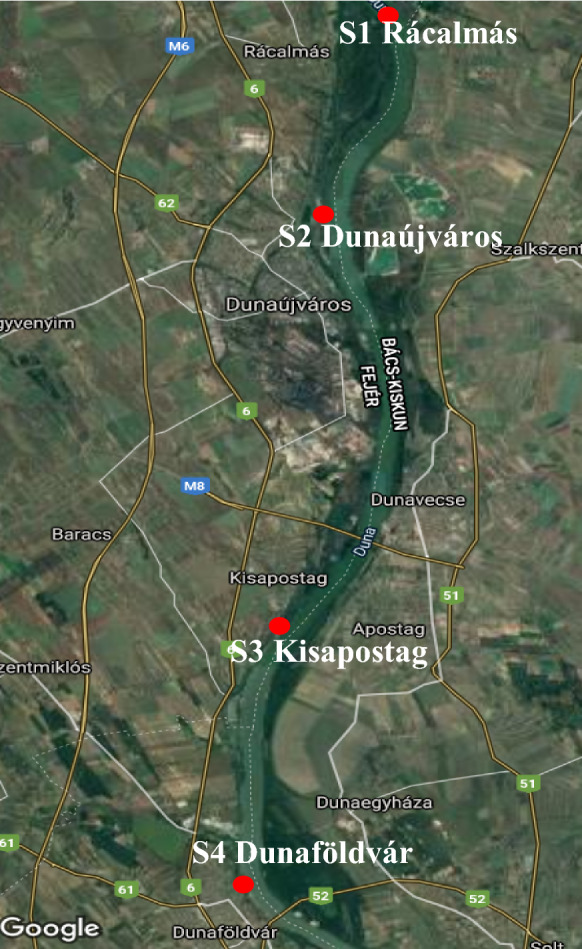
Sampling points (S1–S4) at the bank of the river Danube, near Dunaújváros industrial town, Hungary (*Google Map*)*.* Sites of field experiments were indicated by red points. Geograhical positions of experimental sites are shown in Table 3. S1: Rácalmás Island, S2: Dunaújváros town, S3: Kisapostag, village and S4: Dunaföldvár village, Hungary

For the field experiments, four sampling sites were chosen along the river Danube: S1) Rácalmás, S2) Dunaújváros, S3) Kisapostag and S4) Dunaföldvár (Fig. 1). All sites were located approximately 50 m far from the bank of the river Danube. These areas are usually underwater during the flooding period of the river, so the river can spread its bottom sediment on them occasionally. Table 3 shows the GPS data of the sampling sites. According to the soil map of Hungary (*portal.nebih.gov.hu*) the soil genetic type of these areas is vertisol, at the studied sites we used the alluvial soil with humus.

**Table 3:** GPS data of the four sampling sites, selected for further plant-growing tests in this study. Experimental sites are at close vicinity of river bank, Danube, Hungary

Sampling points	Sites of the samplings	GPS data of sites	
		EOV coordinates	WGS’84 coordinates
S-1	Rácalmás (S1)	EOV (X) 187,512; EOV (Y) 643,574	Lat: N47° 01′ 54,35" Lon: E18° 57′ 46,54"
S-2	Dunaújváros (S2)	EOV (X) 182,834; EOV (Y) 642,178	Lat: N46° 59′ 22,80" Lon: E18° 56′ 40,71"
S-3	Kisapostag (S3)	EOV (X) 171,243; EOV (Y) 641,668	Lat: N46° 53′ 07,40" Lon: E18° 56′ 17,35"
S-4	Dunaföldvár (S4)	EOV (X) 163,565; EOV (Y) 640,541	Lat: N46° 48′ 58,69" Lon: E18° 55′ 24,69"

### Sampling of sediments and plants

Soil/sediment samples were collected from closest vicinity of each test-plants at the beginning and at the end of the experiment in May and July 2017. The pH_(H2O)_ values of the sediments were measured at the sites with a portable pH and soil tester (X4-Life, 4 in 1 Digital pH and soil tester), and also among laboratory conditions. Soil/sediments at four locations were sampled from the upper horizon (0–10 cm). The sampling area was 1 m^2^ in the case of each plants. Composite sub-samples were collected from 5–5 points along the two diagonals of using a stainless steel soil sampler (Pürkhauer 5012 soil sampler). The total weight of the soil/sediment samples was between 790 and 870 g/each individual plants. During transportation, they were stored at 4 °C. In the laboratory, samples were dried in a drying chamber at 105 °C to a constant dry weight. Then sediment samples were ground and sieved to 2 mm particle size. The content of PTE of each sample was determined with XRF (Oxford Instruments, X-MET 5100 X-ray Fluorescence Analyser).

For the field experiment, four types of plant species were used. White mustard (Sinapis alba L.), perennial rye grass (Lolium perenne L.), and two edible vegetable plants, as the radish (Raphanus sativus L. var. Szentesi óriás vaj) and green pea (Pisum sativum L. var Rajnai törpe) were chosen as test plants. Among these plants, white mustard is used in standardised eco-toxicity tests of the soils in Hungary (MSZ 21 976–17:1993 Hungarian Standard). Radish and green pea are edible vegetables, which might result in therefore some potential health risk, when grown in the soils of the industrial town. The planting inner area of the test-plants was 1 m^2^/plot, and 4 replicates were used for the growth tests. The average growing density was 20–30 plants per m^2^ for radish, 30–40 plants per m^2^ for white mustard, 40–60 plants per m^2^ for green pea and 120–150 plants per m^2^ for grass, in a totally randomised arrangement of each replicates and treatments. At the end of the experiment, sediment and plant samples were collected from the studied sites. During the plant sample collection, total plants (roots + stems and leaves) were harvested. Soil/sediment samples were stored at about 4 °C during and after the transportation. The average total green masses of the collected plant samples/sites were 82 g for green pea, 38.6 g for white mustard, 98.7 g for rye grass and 10.5 g for radish. The average total dry masses of the analysed plants/sites were 69.9 g for green pea, 34.8 g for white mustard, 55.8 g for rye grass and 8.9 g for radish. In the laboratory, every plant samples were washed with deionized water to remove any sediment particles and divided into roots and shoots (and also for stems and leaves). Samples were dried for constant weight at 105 °C. Preceding of the instrumental analysis, the dried samples were cut into small pieces for grinding. Heavy metals, PTE were analysed using XRF (Oxford Instruments, X-MET 5100 X-ray Fluorescence Analyser). Every sample was measured by this analyser by 3 times, and the measuring time was 30 s. for each. The level of measured toxic elements in the soil settled river sediment and in test plant samples was compared to the Hungarian regulatory limits (Table 4) of KvVM-EüM-FVM Regulation No 6/2009 and FVM Regulation No 44/2003.

**Table 4:** Average permissible limit values of potentially toxic elements (PTE) in the river Danube sediments and in the biomass of test-plants on the basis of Hungarian regulatory limits. (^*1*^*KvVM-EüM-FVM Regulation No 6/2009, *^*2*^*FVM Regulation No 44/2003, *^*3*^*Simon 2006, *Simon, 2014)

Potentially toxic elements (PTE)	River sediment^1^ (mg/kg)	Shoot biomass^2^ (mg/kg)	Mean in plants in Hungary^3^ (mg/kg)	In plants with toxicity^3^ (mg/kg)
Cadmium and compounds	< 0.08	1	< 0.3–0.5	5–20
Chromium (dissolved)	20	NDA^+^	0.02–0.2	1–10
Lead and compounds	1.2	10	2	30–300
Nickel and compounds	4	NDA^+^	0.1–5	10–100
Copper (dissolved)	10	15	5–20	20–30
Zinc (dissolved)	75	150	25–150	400 <

^+^NDA = No data available

### Data analyses

1. *Pollution load index* (*PLI*)
  The sediment contamination for metals was calculated of using the pollution load index (PLI).
  $${\text{PLI}} = \frac{{c_{{{\text{sediment}}}} }}{{c_{{{\text{reference}}}} }}$$
  where *c*_sediment_ was the metal concentration of the river sediment, *c*_reference_ was the metal concentration of the bottom sediment, which was determined at the site of S4. The metal concentration of loamy (yellow) soil was also used as *c*_reference_. Loamy soil texture is the dominant for the bank of the river Danube at the studied sites. (JDS^3^, ^2015^; Khan et al. 2008)*.*
2. *Bioaccumulation factor* (*BAF*)
  Bioaccumulation factor (BAF) was calculated to determine the ratio of metal concentration in the plant shoots (stems and leaves) to sediment:
  $${\text{BAF}} = \frac{{c_{{{\text{shoots}}}} }}{{c_{{{\text{sediment}}}} }}$$
  where *c*_shoots_ is the total metal concentration in the plant (leaves, stems), and *c*_sediment_ are metal concentrations in the river sediment. The BAFs of the known hyperaccumulator plants are generally greater than 1 (Lago-Vila et al. 2015; Mehr et al. 2020)*.*
3. *Translocation factor* (*TF*)
  Metal concentrations in the sediment and plant samples were calculated on a dry weight basis. The translocation factor (TF) was estimated as the ratio between the metal concentration (mg/kg) in shoots of the test plants (C_shoots_) to the roots (*c*_roots_):
  $${\text{TF}} = \frac{{c_{{{\text{shoot}}}} }}{{c_{{{\text{root}}}} }}$$
  where *c*_shoot_ were calculated from the metal concentration of the plant-parts, grown above the surface. When TF > 1, it shows that the metals were translocated from roots to shoots efficiently (Baker & Brooks, 1989; Khan et al. 2008; Lago-Vila et al. 2015; Mehr et al. 2020)*.*
4. *Daily intake of metals* (*DIM*)
  The daily intake of metals (DIM) was calculated by the following equation (Khan et al. 2008):
  $${\text{DIM}} = \frac{{C_{m} \times C_{f} \times D_{i} }}{{B_{w} }}$$
  where *C*_*m*_ = total metal concentration in test plants (mg/kg) on dry weight basis, *C*_*f*_ = the fresh to dry weight conversion factor for vegetables, *D*_*i*_ = daily intake of green vegetables (g/person/day), *B*_*w*_ the average body weight (kg) for Hungarian adults and children (Rattan et al., 2005). The conversion factor was 0.085 for every plant (Rattan et al. 2005).
5. *Health risk index* (*HRI*)

The risk for human health was determined for intake of Cd, Ni, Cu and Zn through consumption of radish and green pea by the following equation (Khan et al. 2008).

$${\text{HRI}} = \frac{{{\text{DIM}}}}{{{\text{RfD}}}}$$

The Health Risk Index (HRI) for the locals through the consumption of contaminated vegetables was assessed based on the food chain and the reference oral dose (RfD) for each metal. The HRI < 1 means the exposed population is assumed to be safe (Mehr et al. 2020; Rattan et al. 2005).

## Results and Discussions

1. Physical and chemical characteristics of studied soil/sediment samples

Among the known soil-physical and -chemical characteristics of studied soil/sediment samples, the following characteristics were examined, as suggested by Buzás (1988): porosity, sand/silt/clay ratio is 5/41/54%, pH_(H2O) =_ 7.06, CaCO_3_ = 2.51%, hydrolysable N = 10 mg/100 g, AL-K_2_O = 106 mg/100 g, AL-P_2_O_5_ = 18,0 mg/100 g, average organic matter content by Tyurin is H = 4.8%, Arany type of plasticity index K_A_ = 67.4, Munsell type of colour, as dry is 10YR6/1, as wet is 10YR5/1 at the representative vertisol in the Dunaujváros region, in Hungary.

**Fig. 2:**
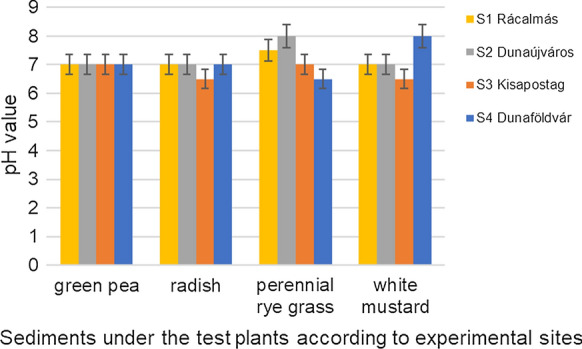
Measured pH values of the studied soil/sediment samples, originating from four sampling sites along the river Danube, in close vicinity of an industrial town Dunaújváros, Hungary. Samples were collected from the rhizosphere of four different test-plants used in the studies. Data are means of 4 replications, bars indicate standard deviation of the data

The pH _(H2O)_ values of the soil/sediment samples, after the growth of test plants were measured at each sampling points. Figure 2 represents the average pH values of the collected soil/sediment samples from each experimental site. The lowest pH value was 6.5, the highest result was found to be 8,0. The average pH value of the soil/sediment samples at S1 site was 7.12, at S2 site was 7.25, at S3 site was 6.75 and at S4 site was 7.12. The average pH value of each experimental site was 7.06. In this case, the sediment was considered to be non-acidic, mainly near to the level of neutral. Considerable differences between pH data were not observed.

**Fig. 3:**
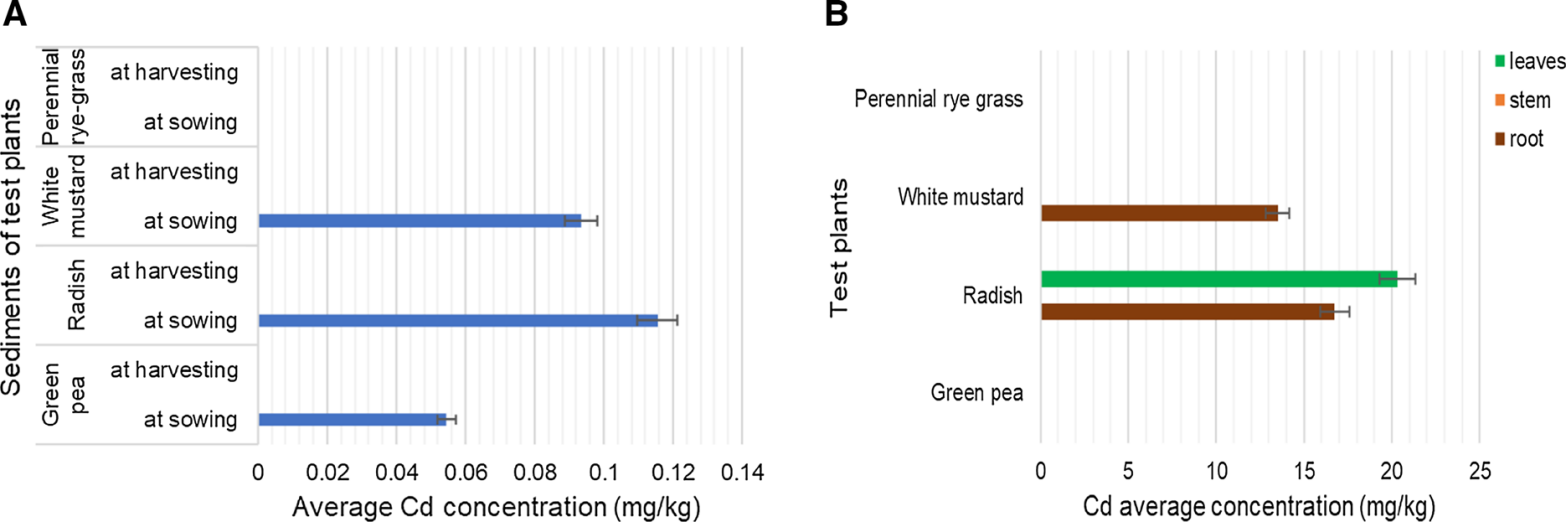
Average cadmium (Cd) concentration of samples. **A** -in the river soil/sediments at sowing (blue) and harvesting (green, no data), as a function of four different test-plants. **B** -in the roots (brown), stems (orange, no data) and the leaves (green) biomass of the studied test-plants. Data are means of 4 replications; bars indicate standard deviation of the data.

**Fig. 4:**
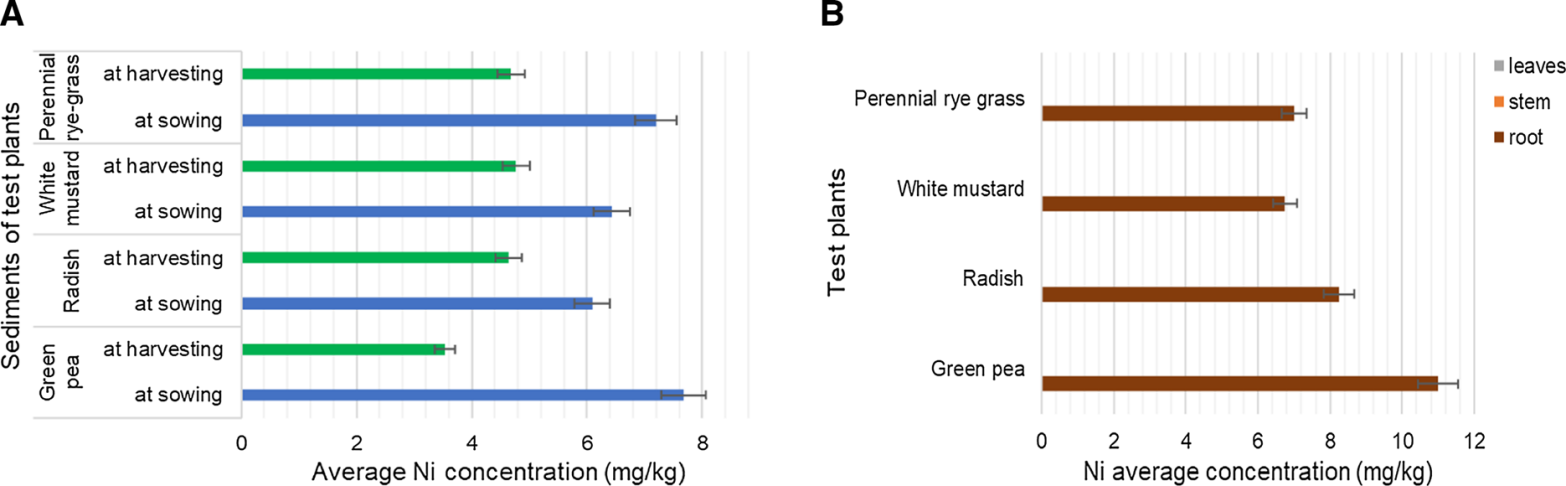
Average nickel (Ni) concentration of samples. **A** -in the river sediments at sowing (blue) and harvesting (green) as a function of four different test-plants. -in the roots (brown), stems (orange, no data) and leaves (green, no data) biomass of the studied test-plants. Data are means of 4 replications; bars indicate standard deviation of the data

**Fig. 5:**
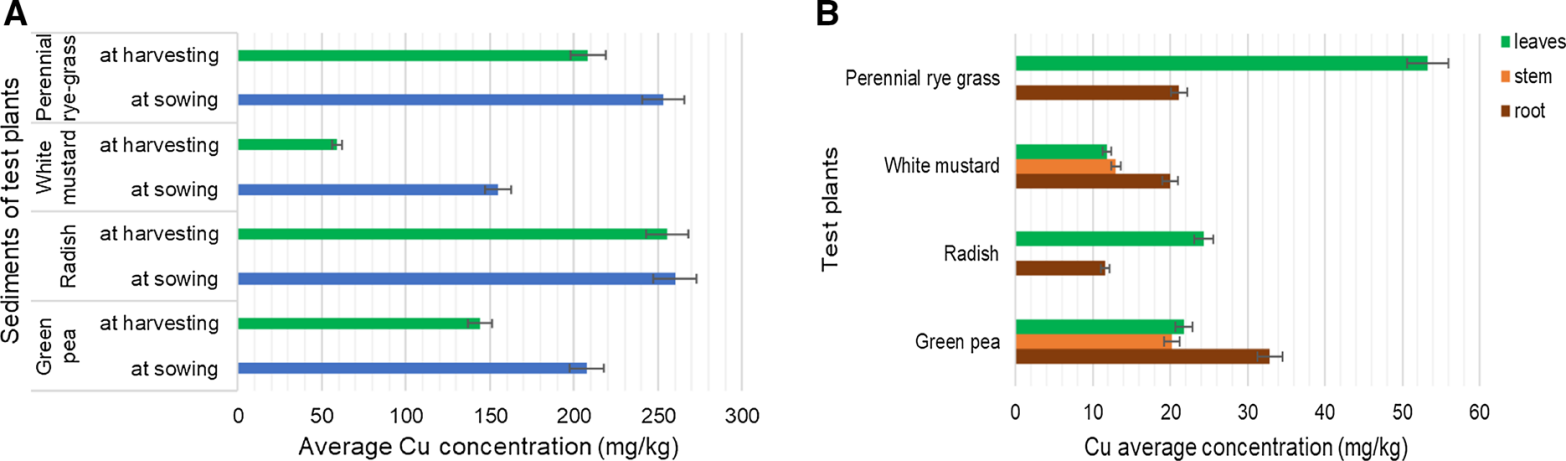
Average copper (Cu) concentration of samples. **A** -in the river sediments at sowing (blue) and harvesting (green) as a function of four different test-plants. **B** -in the root (brown), stem (orange) and leaves (green) biomass of the studied test-plants. Data are means of 4 replications; bars indicate standard deviation of the data

**Fig. 6:**
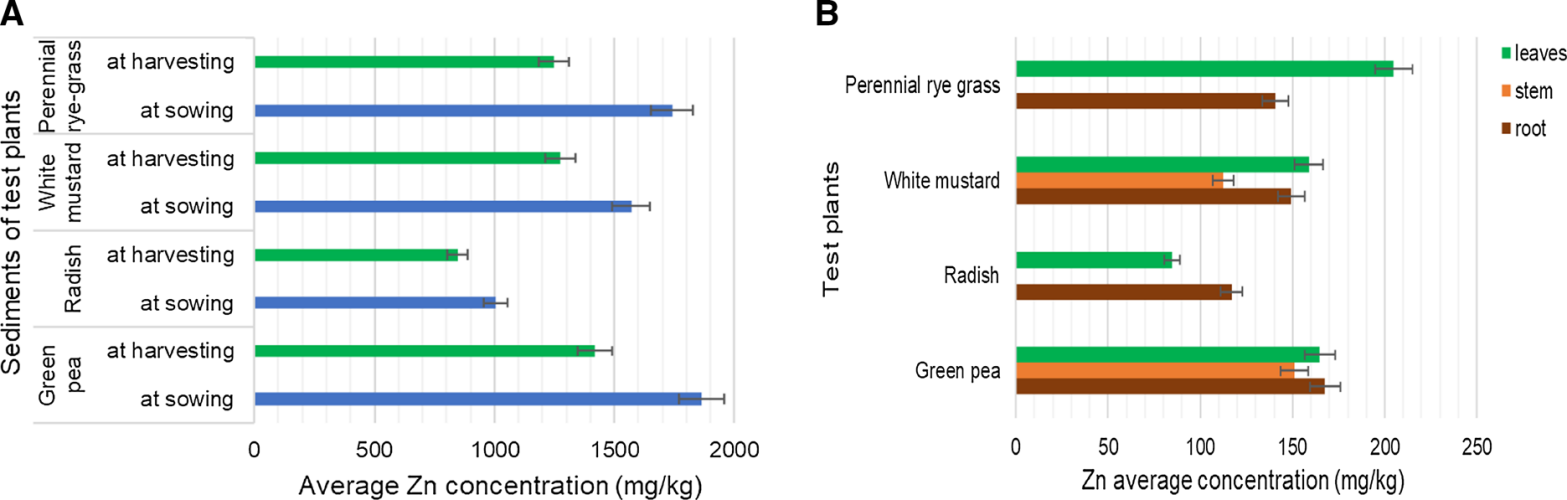
Average zinc (Zn) concentration of samples. **A** -in the river sediments at sowing (blue) and harvesting (green) as a function of four different test-plants. **B** -in the root (brown), stem (orange) and leaves (green) biomass of the studied test-plants. Data are means of 4 replications; bars indicate standard deviation of the data

2. Content of potentially toxic elements in tested plant parts

- Cadmium accumulation
  Based on Fig. 3a, the average Cd concentration of the sediment samples was between 0.05 and 0.11 mg/kg, assessed at the beginning of the experiment. The Cd concentrations of the sediment samples did not exceed of the Hungarian regulatory level, which is 1 mg/kg (*KvVM-EüM-FVM Regulation No 6/2009*). It was also lower than the probable effect concentration (PEC) value of 4.98 mg/kg (MacDonald et al. 2000) and the JDS 3 value of 0.65 mg/kg for bottom sediment. Cadmium was found to be non-accumulated from the sediment in case of the perennial rye grass. Compared to the Cd concentrations of the sediments, Cd was not accumulated from the sediments either after finishing of the experiment.
  Between test plants only white mustard and radish could accumulate cadmium from the contaminated soil (Fig. 3b). Cd was detected only in the roots of white mustard, against radish which could translocate this element to the leaves. The cadmium concentration was between 13 and 20 mg/kg in the parts of test plants which is higher than the Hungarian average value of 0.5 mg/kg (Simon, 2014). The Cd concentration of the plants was in the range of 5–20 mg/kg, this level could be toxic for the plant (Simon, 2006, 2014; Szegedi, 2011). The result of perennial rye grass was lower than the results found by Bidar et al. (2007). The Cd concentration of radish was higher than that of radish found by Davis (1992) and Khan et al. (2008)*,* but lower than results found by Marchiol et al. (2004). The Cd concentration of green peas was lower than results found by Oloruntoba et al. (2017) *and* Płaza et al. (2019). The Cd value of white mustard was higher than the results found by Fargašová (2001).

- Nickel accumulation
  The average Ni concentrations of the sediment samples were between 3.5 and 7.7 mg/kg (Fig. 4a). The concentrations from sowing to harvesting were found to decrease. The concentrations were lower than the Hungarian regulatory limit of 40 mg/kg (*KvVM-EüM-FVM Regulation No 6/2009*) and also were lower than the PEC value of 48.6 mg/kg for Ni (MacDonald et al. 2000) or the JDS3 (*Joint Danube Survey 2015*) value of 10 mg/kg for bottom sediment.
  Among the main organs of the test plants, roots could accumulate Ni, and it was not translocated to shoots or leaves (Fig. 4b). Among the test plants green pea could accumulate Ni in the highest rate (11 mg/kg) and white mustard in the lowest rate (6.75 mg/kg). The average values for the Ni concentration of the plants in Hungary are between 0.1and 5 mg/kg. The average Ni concentrations of test plants exceeded this limit, they were between 6 and 11 mg/kg. The limit of toxicity is around 10–100 mg/kg for plants. The results did not exceed this range (Simon, 2006; Szegedi, 2011)*.* The result of radish was lower than the results found by Rattan et al. (2005)*,* Marchiol et al. (2004)*,* and was higher than results found by Khan et al. (2008). The Ni concentration of green peas was higher than result measured by Oloruntoba et al. (2017) and Płaza et al. (2019).

- Copper accumulation
  Based on Fig. 5a, the Cu concentration was higher in the sediment samples than the Hungarian regulatory level of 75 mg/kg (*KvVM-EüM-FVM Regulation No 6/2009*) or the PEC value of 149 mg/kg (MacDonald et al. 2000) or the JDS 3 (*Joint Danube Survey 2015*) maximum value of 101.1 mg/kg for bottom sediment*.* The average Cu concentrations of the sediments of the test plants were between 60 and 260 mg/kg. The sediments of white mustard had the lowest copper concentration (59 mg/kg). The change of the concentrations of Cu from sowing to harvesting was the smallest (1.7%) in the case of radish.
  Copper belongs to the group of essential elements; the test plants therefore were able to uptake or translocate it in relatively high levels. Regarding of the different studied parts of the test plants, the roots of green pea and white mustard were found to contain the Cu in high levels (Fig. 5b) and these plants could translocate this element into their stems or leaves. The Cu contaminations of the plant samples were similar to the Hungarian average value of 5–20 mg/kg. The roots of green pea and perennial rye grass and the leaves of radish and perennial rye grass had higher copper concentration than 20 mg/kg, which could have toxic effect on the shoots (Simon, 2006, 2014; Szegedi, 2011). The Cu concentration of radish was higher than the results found by Davis (1992) and Khan et al. (2008) and was comparable to results found by Rattan et al. (2005)*.* The Cu concentration of green peas was comparable with results found by Oloruntoba et al. (2017) and higher than the results by Płaza et al. (2019). The copper concentration of the parts of the white mustard was higher than the results found by Fargašová (2001).

- Zinc accumulation
  The Zn concentration of the sediment samples was in average 1370 mg/kg, which extremely exceeded the Hungarian regulatory level, which is 200 mg/kg (*KvVM-EüM-FVM Regulation No 6/2009*)*,* the PEC value of 459 mg/kg (MacDonald et al. 2000) and the JDS3 (*Joint Danube Survey 2015*) maximum value of 198 mg/kg for bottom sediment. Based on Fig. 6a, the Zn concentration of the sediments of the test plants was 1000–1800 mg/kg before the experiment, at the end of the experiment it was 840–1420 mg/kg. The concentration of this element decreased from the sowing to the harvesting of the test plants.
  Zinc is also known as an essential element. According to Fig. 6b, the higher Zn concentrations (80–200 mg/kg) were measured mainly from the leaves of the test plants. The concentration of the Zn element exceeded the average Hungarian value which is 25–150 mg/kg, but not higher than the tolerable value, which is 400 mg/kg (Simon, 2006, 2014; Szegedi, 2011). Among the test plants, the white mustard and the green pea could accumulate this element in higher rates; the Zn was detected in each part of these two plants. This result is lower than the value found by Bidar et al. (2007) for the roots and comparable with the results for shoots of perennial rye grass. The zinc concentration of radish was lower than results found by Davis (1992)*,* and Marchiol et al. (2004)*,* and higher than results found by Rattan et al. (2005) and Khan et al. (2008). The zinc concentration of green peas was higher than results found by Oloruntoba et al. (2017) and Płaza et al. (2019). The Zn concentration of the white mustard was higher than the results found by Fargašová (2001).

3. Pollution Load Index (PLI) of studied potentially toxic elements
  For the calculations of pollution load index (PLI), two references were used. One is the concentrations of the bottom sediment by JDS 3 (*Joint Danube Survey 2015*), other is the average metal concentration of loamy soil, which is the dominant soil type around the sampling sites. The calculations of PLI were done in the case of the beginning and at the finishing of the experiment. The studied river sediments were more polluted for Cu and Zn, in comparison with the reference sediment or soil (originating from non-industrialised site). In the sediment samples, metal concentrations were significantly higher in the case of copper and zinc compared with the reference bottom sediment or loess soil (Table 5). The PLI values for these two elements were higher than 1 (Cu: 9.53 and 12.5; Zn: 10.83 and 13.98). In Table 5 “pre” means the beginning, “post” is the finishing of the experiment.

**Table 5:** Pollution load index (PLI) values for sediment samples preceeding (pre) and finishing (post) of the experiment, calculated by 2 different ways and compared it to the control of sediments and of the reference samples: (1) Average content of potentially toxic elements (PTE) in bottom sediment of site JDS 3 (*Joint Danube Survey 2015*), (2) Content of PTE in dominant loamy soil of bank of the river Danube, Hungary

PTE	C_sediment_ (mg/kg)		C_reference_^1^ (mg/kg) (JDS 3)	C_reference_ (mg/kg) (Loamy soil)	PLI^1^ (JDS 3)		PLI (Loamy soil)	
	Pre	Post			Pre	Post	Pre	Post
Cd	0.07	0.00	0.95	46.5	0.07	0.00	0.00	0.00
Ni	6.85	4.41	20	15	0.34	0.22	0.46	0.29
Cu	218.79	166.75	17.5	11	12.50	9.53	19.89	15.16
Zn	1544.35	1196.78	110.5	39	13.98	10.83	39.60	30.69

4. Bioaccumulation (BAF) and translocation factor (TF) of studied potentially toxic elements
  From the average metal concentration of the sediment and the plants samples, TF and BAF factors were also calculated (Table 6), as suggested by Al-Hwaiti and Al-Khashman (2015), Lago-Vila et al. (2015) and Mehr et al. (2020)*.* Overall, it was realised, that the BAF factor for Cd, Ni, Cu and Zn was less, than 1, only the TF factor of Cu and Zn was higher than 1.
  For zinc and copper, green pea and white mustard can be considered as a highly accumulating plant. The trend of BAF for toxic metals was in the order of Cu > Zn > Cd, Ni. Because of BAF values were less than 1, test plants do not belong to the hyperaccumulator species. Taking all the test plants together, relative orders of TF from the roots to shoots were Cu > Zn > Cd > Ni. These results show that Cu and Zn have the greatest potential, followed by Cd and Ni. Based on the TF values, the efficiency of the test plants to translocate the studied metals from the roots to their shoots could be arranged in the following order:
  *Cadmium* radish > white mustard and green pea and perennial rye grass. Among the test plants only radish could translocate Cd to the upper parts.
  *Nickel* No test plant could translocate this element from the roots to the shoots.
  *Copper* perennial rye grass > radish > green pea > white mustard.
  *Zinc* green pea > white mustard > perennial rye grass > radish.
  This information can be very useful in selecting the suitable test plants to be grown on metal-contaminated river sediments.

**Table 6:** Bioaccumulation factor (BAF) and translocation factor (TF) of some potentially toxic elements for the tested plants of green pea, radish, white mustard and perennial rye grass, grown in the sediments of the river Danube near an industrial town of Dunaújváros, Hungary (*n* = 4)

Test plants	BAF^+^				TF^++^			
	Cd	Zn	Cu	Ni	Cd	Zn	Cu	Ni
Green pea (*Pisum sativumi*	0	0.223	0.291	0	0	1.883	1.277	0
Radish (*Raphanus sativus*)	0	0.1	0.095	0	1.214	0.722	2.093	0
White mustard (*Sinapis alba*)	0	0.213	0.421	0	0	1.816	1.242	0
Perennial rye grass (*Lolium perenne*)	0	0.164	0.256	0	0	1.456	2.525	0

^+^BAF = ratio between the metal concentration of shoots and sediment; ^++^TF = ratio between the metal concentration of the shoots and roots

5. Risks associated with green pea and radish

The average metal concentration of the test plants samples DIM (Daily Intake of Metals) and HRI (Health Risk Index) was also calculated (Table 7). These calculations were made for these edible vegetables, green pea and radish.

**Table 7:** Calculated health risk index (HRI) and daily intake (DIM) of potentially toxic elements in the edible parts of the test plants, established for adults and for children

Testplants	DIM^+^ (adults)	DIM^+^ (children)	HRI^++^ (adults)	HRI^++^ (children)
Cd				
*Green pea*	0	0	0	0
*Radish*	0.0083	0.0208	8.34	20.77
Ni				
*Green pea*	0.0025	0.0062	0.12	0.31
*Radish*	0.0019	0.0046	0.09	0.23
Zn				
*Green pea*	0.1087	0.2709	0.36	0,9
*Radish*	0.0453	0.1129	0.15	0.38
Cu				
*Green pea*	0.0168	0.0419	0.42	1.05
*Radish*	0.0081	0.0201	0.2	0.5

The plants of green pea (*Pisum sativum L.* var. Rajnai törpe) and radish (*Raphanus sativus L.* var. Szentesi óriás vaj) were grown in the sediments near the river Danube, Dunaújváros, Hungary (*n* = 4)

^+^DIM = (Multiplicity of total metal concentration in test plants on dry weight basis and fresh to dry weight conversion factor for vegetables and Daily Intake of green vegetables)/the average body weight (kg) for Hungarian adults and children; ^++^HRI = Ratio of daily intake of metals and the reference oral dose for metals

The daily intake of metals (DIM) values (0—0.27) for the studied metals were not high, all of them were less than 1. The highest intakes of Zn were from the consumption of green pea and radish for both adults and children.

The HRI values of children were higher than 1 for Cd in the case of radish (*20.77*), and for Zn and Cu in the case of green pea (*Zn: 0.9; Cu: 1.05*). Most of the HRI values were less than 1 for adults. Therefore, these green vegetables are not likely to induce any health hazard to adult consumers. The HRI values for children suggested that the consumption of these plants grown in contaminated sediments can result in some potential risks more particularly for the children.

## Conclusion

In this study, some of the potentially toxic elements (PTE), the Cd, Ni, Cu and Zn levels of the sediment-plant samples were studied. Selected test-plants were grown in the sediments of the occasionally flooded area of the river Danube. The sediments in sampling points S3 and S4 might be potentially contaminated, as a result of the severe industrial activity in Dunaújváros town, Hungary. The metal uptake, the mobilisation potential of the measured elements was determined to get information about the phytoremediation potential and the health risk of the studied test plants on the site. Among the analysed Cd, Ni, Cu and Zn concentrations, the copper and zinc were found to be above the Hungarian standard limits in the river sediment. For this reason, the pollution load index (PLI) values for these two elements were higher than 1 in general. The studied river sediments were more polluted for Cu and Zn than the reference bottom sediment (*JDS 3*) or reference loess soil, equally before and after the plantation. Metal concentration of each sediment samples decreased up till the end of the experiment. Ratio of the reduction was highly dependent on the plant species. It was between 35–66 % for Ni, 2–62 % for Cu and 16–28 % for the Zn. The highest reduction ratio was observed in the sediment with green pea, for Ni (66%), with white mustard for Cu (62%) and with perennial rye grass for Zn (28%). Plant samples were found to be highly polluted with cadmium, copper and zinc elements, in comparison with the sediment samples. Concentration of each analysed elements was above the average Hungarian limits. BAF factor for Cd, Ni, Cu and Zn was less than 1. Test plants were not able to accumulate the studied toxic metal elements in high rate from the sediments into their tissues. The BAF values for toxic metals were in the order of Cu > Zn > Cd > Ni for each plant, studied. The TF values from the river sediment to all the test plants grown on it were the same as BAF values, Cu > Zn > Cd > Ni. Among the test plants, only the radish could translocate Cd into the leaves. In the case of Cu and Zn, the TF values of each plant species were more than 1. These metals are essential elements, so they can move easier to the aboveground organs (shoot) parts of the plants. In addition to BAF and TF values, the daily intake of metals (DIM) and health risk index (HRI) were also determined for the edible, cultivated plants. The DIM values for the studied metals were not high, all of them were less than 1. The HRI values were less than 1 for adults in the case of every test plants, but HRI values for children were more than 1 in the case of Cd in radish, and Cu, Zn in green pea*.* Therefore, the consumption of these green plants grown in the studied sediments might result in some risks for the children. Further research is necessary to find other appropriate test-plants with more efficient bioaccumulation potential so as to reduce of the high risk of studied toxic elements.

## Data Availability

The datasets generated during and/or analysed during the current study are available from the corresponding author on reasonable request.

## Code availability

No any special code was applied in the study.
